# Health at Every Size®-Based Interventions May Improve Cardiometabolic Risk and Quality of Life Even in the Absence of Weight Loss: An Ancillary, Exploratory Analysis of the Health and Wellness in Obesity Study

**DOI:** 10.3389/fnut.2022.598920

**Published:** 2022-02-22

**Authors:** Mariana Dimitrov Ulian, Ana Jéssica Pinto, Priscila de Morais Sato, Fabiana B. Benatti, Patricia Lopes de Campos-Ferraz, Desire Coelho, Odilon J. Roble, Fernanda Sabatini, Isabel Perez, Luiz Aburad, André Vessoni, Ramiro Fernandez Unsain, Marcelo Macedo Rogero, Geni Sampaio, Bruno Gualano, Fernanda B. Scagliusi

**Affiliations:** ^1^Department of Nutrition, School of Public Health, University of São Paulo, São Paulo, Brazil; ^2^Applied Physiology & Nutrition Research Group, University of São Paulo, São Paulo, Brazil; ^3^School of Applied Sciences, State University of Campinas, Limeira, Brazil; ^4^Faculty of Physical Education, State University of Campinas, Campinas, Brazil; ^5^Food Research Center (FoRC), CEPID-FAPESP, Research Innovation and Dissemination Centers São Paulo Research Foundation, São Paulo, Brazil

**Keywords:** obesity, lifestyle intervention, cardiovascular risk, physical activity, weight-neutral approach

## Abstract

We examined whether weight loss following HAES®-based interventions associates with changes in cardiometabolic risk factors and quality of life of women with obesity. This was an exploratory, ancillary analysis of a 7-month, mixed-method, randomized controlled trial. Fifty-five women (age: 33.0 ± 7.2; BMI: 30–39.9 kg/m^2^) were included in this study. Body weight, cardiovascular risk factors, clustered cardiometabolic risk, and quality of life were assessed before (Pre) and after HAES®-based interventions (Post). Delta scores (Post-Pre) were calculated for each outcome and used in linear regression models. After adjusting by potential confounders, weight loss was associated with improvements in waist circumference (β = 0.83, *p* <0.001), fasting glycemia (β = 0.45, *p* = 0.036), total cholesterol (β = 1.48, *p* = 0.024), LDL (β = 1.33, *p* = 0.012), clustered cardiometabolic risk (β = 0.18, *p* = 0.006), and quality of life (β = −1.05, *p* = 0.007). All participants but one who reduced body weight (*n* = 11) improved clustered cardiometabolic risk and quality of life. Of relevance, 34% and 73% of the participants who maintained or gained weight improved clustered cardiometabolic risk and quality of life, respectively, although the magnitude of improvements was lower than that among those who lose weight. Improvements in cardiovascular risk factors and quality of life following HAES®-based interventions associated with weight loss as expected. However, most of the participants who maintained or even gained weight experienced benefits to some extent. This suggests that weight-neutral, lifestyle-modification interventions may improve wellness and health-related outcomes, even in the absence of weight loss.

## Introduction

Intentional weight loss remains as the cornerstone treatment of people with obesity (1). However, it has been suggested that the monolithic focus on weight loss as the only determinant of success for strategies that aim to manage obesity may preclude opportunities to focus on lifestyle behaviors. These behaviors are associated with benefits across a wide range of health outcomes, regardless of weight status or weight change (2).

Diet- and exercise-induced weight loss are knowingly associated with reduced cardiometabolic risk (3). However, evidence suggests that people with obesity engaged in non-restrictive diets and exercise interventions may also exhibit improvements in abdominal circumference, insulin resistance, dyslipidemia, systemic inflammation, hypertension, and all-cause mortality with or without weight loss (4). Exercise reduces waist circumference and visceral fat, which is *per se* associated the improvements in cardiovascular risk factors independently of body weight changes (3, 5, 6).

The Health at Every Size® (HAES®) approach promotes a shift from a weight-centered to a weight-neutral approach by encouraging people with different body sizes to engage in healthier behaviors, with no primary focus on losing weight (7). Its principles include the promotion of a pleasurable and sustainable physical activity practice, and flexible, individualized eating based on hunger, satiety, nutritional needs, and pleasure. We recently showed that an intensive HAES®-based intervention was capable of improving participants' eating attitudes and practices, perception of body image, cardiorespiratory fitness, physical function, and health-related quality of life (8, 9). The central aim of our physical activity program was to increase enjoyment and autonomy in daily physical activities; thus, the participants were encouraged to exercise at a self-selected intensity. The nutritional intervention was based on nutritional counseling and diets were not prescribed. Instead, participants were encouraged to eat based on the principles of the HAES® approach. To date, it remains unclear whether HAES® interventions with the above-mentioned features can yield health-related benefits irrespective of changes in body weight. In this ancillary, exploratory study, we examined whether weight loss following a HAES®-based intervention is a determinant of changes in cardiometabolic risk factors and quality of life in women with obesity. Our working hypothesis was that weigh loss would relate to improvements in cardiometabolic health and wellness as expected, but even those participants who did not lose weight would experience some beneficial effects from the interventions.

## Methods

### Study Design and Participants

The ancillary analysis is derived from a 7-month, mixed-method, randomized controlled trial. This study was conducted according to the guidelines laid down in the Declaration of Helsinki and all procedures involving human subjects were approved by the Ethics Committee of the School of Public Health, University of São Paulo (protocol 1.738.855). Written informed consent was obtained from all participants. This study is registered at clinicaltrials.gov (NCT02102061). Details regarding the experimental design, intervention, measures and outcomes, and main results can be found elsewhere (8, 10). In brief, the trial was designed to test the efficacy of two HAES®-based interventions of different intensities on health- and wellness-related variables in obese women. The intensive HAES® group underwent a program comprising three-times-a-week physical activity sessions, bimonthly individual nutritional sessions, and five philosophical workshops. The traditional HAES® group (control) only attended bimonthly educational lectures based on the HAES® principles. In this ancillary analysis, data from 55 participants who had complete body weight data were analyzed. We opted for combining data from both groups (intensive HAES® *n* = 36; traditional HAES® *n* = 19) to increase the power of our analysis, after considering that the assessment of separate groups would not significantly add to the current research question. Changes in anthropometric measures, cardiovascular risk factors, clustered cardiometabolic risk, and quality of life (delta score) were calculated and associated with weight loss.

### Anthropometric Measures

Weight was measured by a digital scale. Weight loss was defined as a decrease in body weight ≥3%, in accordance with the definition of weight maintenance proposed by Stevens et al. (11). Waist circumference was measured using a plastic tape measure placed in the smallest circumference between the lowest margin of the ribs and the upper margin of the iliac crest with subjects standing.

### Cardiovascular Risk Factors and Clustered Cardiometabolic Risk

Cardiovascular risk factors included blood pressure, fasting plasma glucose, insulin, glycated hemoglobin, and lipid profile. Homeostatic Model Assessment (HOMA-IR) was also calculated. Glucose was assessed using a colorimetric enzymatic assay (Bioclin, Brazil). Insulin was assessed using a radioimmunoassay technique (Diagnostic Products Corporation, Inc). Lipid profile was assessed using enzymatic colorimetric assays (CELM, São Paulo, Brazil).

Continuous clustered cardiometabolic risk was computed using waist circumference, mean blood pressure (average of systolic and diastolic pressure), fasting plasma triglycerides, high-density cholesterol (HDL), and glucose (12). Reference values were 88 cm, 115 mmHg, 150, 50, and 100 mg/dL, respectively. All variables were standardized [*z* = (value – reference) / SD]; for HDL (protective for cardiometabolic risk), *z*-score was inverted. The risk score was the sum of all standardized scores, with higher *z*-scores indicating higher cardiometabolic disease risk.

### Quality of Life

Quality of life was assessed by means of the total score of the World Health Organization Quality of Life—BREF questionnaire (WHOQOL-BREF), which has been translated to Portuguese and validated for the Brazilian population (13, 14). Higher scores represent higher quality of life, and the calculations were made following the syntax proposal by The WHOQOL Group (15). A total score of the WHOQOL-BREF was calculated. Such score consists of calculating the arithmetic mean of the scores of the 26 questions of the instrument for each participant (15).

### Statistical Analysis

Deltas score (Post-Pre) was calculated for the dependent variables to assess changes following the interventions. Linear regression models were used to test possible associations between changes in body weight (independent variable) and changes in waist circumference, cardiovascular risk factors, clustered cardiometabolic risk, and quality of life (dependent variables). Regression models were unadjusted or adjusted by potential confounding factors (i.e., age, body mass index, and baseline value of the dependent variable). Cohen's d effect sizes (ES) were calculated for changes in clustered cardiometabolic risk and quality of life for participants who lost (*n* = 11), maintained or gained body weight (*n* = 44), and for participants who maintained or gained body weight and improved clustered cardiometabolic risk (*n* = 15) and quality of life (*n* = 32). Data analysis was performed using the SAS (9.3) for Windows. The level of significance was set at *p* ≤ 0.050. Data are presented as mean ± SD and β or ES (95% confidence interval [95%CI]), except when stated otherwise.

## Results

Participants' age and BMI were 33.0 ± 7.2 years and 33.6 ± 2.8 kg/m^2^, respectively. Table 1 shows baseline data and delta scores for body weight, waist circumference, cardiovascular risk factors, clustered cardiometabolic risk, and quality of life.

**Table 1:** Baseline values and delta changes for anthropometric measures, cardiovascular risk factors, clustered cardiometabolic risk, and quality of life.

	**Pre**	**Post-to-pre changes**
Body weight (kg)	90.5 ± 10.7	0.2 (−0.9, 1.3)
Waist circumference (cm)	109.0 ± 9.1	−1.6 (−3.9, 0.4)
Glucose (mg/dL)	85.4 ± 11.2	−1.5 (−2.9, 0.4)
Insulin (μU/ml)	18.1 ± 9.5	−2.8 (−4.3, −0.1)
Glycosylated hemoglobin (%)	5.2 ± 0.3	0.1 (0.0, 0.1)
HOMA-IR	3.8 ± 2.2	−0.6 (−1.1, 0.1)
**Lipid profile**		
Total cholesterol (mg/dL)	191.0 ± 34.2	−1.4 (−8.5, 4.3)
HDL (mg/dL)	52.8 ± 16.2	−0.4 (−2.1, 3.4)
LDL (mg/dL)	114.9	−1.9 (−8.9, 1.7)
VLDL (mg/dL)	23.0 ± 10.8	1.2 (−1.3, 3.5)
Triglycerides (mg/dL)	118.0 ± 60.1	2.9 (−11.7, 16.3)
Mean arterial pressure (mmHg)	97.4 ± 8.3	−1.1 (−3.7, 2.2)
Cardiovascular risk (*z*-score)	−1.8 ± 2.8	−0.1 (−0.6, 0.4)
Quality of life	56.3 ± 11.2	7.7 (5.1, 11.6)

*Data presented as mean ± SD or mean (95% confidence interval). HDL, high-density lipoprotein; HOMA-IR, homeostatic model assessment; LDL, low-density lipoprotein; VLDL, very-low-density lipoprotein*.

Weight loss was associated with reductions in waist circumference (β = 0.79, *p* = 0.002), fasting glucose (β = 0.45, *p* = 0.036), L DL (β = 1.54, *p* = 0.018), clustered cardiometabolic risk (β = 0.20, *p* = 0.003), and quality of life (β = −0.82, *p* = 0.039) (Table 2). After adjusting by potential confounders, all associations were maintained; in addition, weight loss was associated with improvements in total cholesterol (β = 1.48, *p* = 0.024) (Table 2). No associations were found between weight loss and other risk factors (all *p* ≥ 0.050).

**Table 2:** Associations between changes in body weight (predictor variable) and waist circumference, cardiovascular risk factors, clustered cardiometabolic risk, and quality of life.

	Model^*^	**β (95%CI)**	***p*-value**
Waist circumference (cm)	Unajust.	**0.79 (0.32, 1.27)**	**0.002**
	Adjust.	**0.83 (0.42, 1.24)**	**<0.001**
Glucose (mg/dL)	Unajust.	**0.45 (0.03, 0.88)**	**0.036**
	Adjust.	**0.45 (0.03, 0.88)**	**0.036**
Insulin (μU/ml)	Unajust.	0.28 (−0.28, 0.83)	0.318
	Adjust.	0.23 (−0.19, 0.65)	0.279
Glycosylated hemoglobin (%)	Unajust.	0.01 (−0.001, 0.03)	0.073
	Adjust.	0.01 (−0.003, 0.03)	0.133
HOMA-IR	Unajust.	0.06 (−0.06, 0.19)	0.319
	Adjust.	0.06 (−0.03, 0.16)	0.200
**Lipid profile**
Total cholesterol (mg/dL)	Unajust.	0.94 (−0.66, 2.54)	0.245
	Adjust.	**1.48 (0.21, 2.74)**	**0.024**
HDL (mg/dL)	Unajust.	−0.66 (−1.34, 0.03)	0.059
	Adjust.	0.05 (−0.51, 0.61)	0.855
LDL (mg/dL)	Unajust.	**1.54 (0.30, 2.79)**	**0.018**
	Adjust.	**1.33 (0.31, 2.36)**	**0.012**
VLDL (mg/dL)	Unajust.	0.04 (−0.56, 0.64)	0.896
	Adjust.	0.04 (−0.54, 0.61)	0.078
Triglycerides (mg/dL)	Unajust.	0.40 (−3.07, 3.86)	0.819
	Adjust.	0.36 (−2.67, 3.39)	0.811
Mean arterial pressure (mmHg)	Unajust.	0.47 (−0.25, 1.20)	0.195
	Adjust.	0.34 (−0.31, 1.00)	0.299
Cardiovascular risk (*z*-score)	Unajust.	**0.20 (0.07, 0.32)**	**0.003**
	Adjust.	**0.18 (0.06, 0.31)**	**0.006**
Quality of life	Unajust.	**−0.82 (−1.59**, **−0.04)**	**0.039**
	Adjust.	**−1.05 (−1.81**, **−0.30)**	**0.007**

*Data presented as unstandardized β coefficient (95% confidence interval)*.

* *“unajust.” is the unadjusted model and “adjust.” is the adjusted model by age, body mass index, and baseline values*.

*Bolded values indicate statically significant (p ≤ 0.05) associations*.

*HDL, high-density lipoprotein; HOMA-IR, homeostatic model assessment; LDL, low-density lipoprotein; VLDL, very-low-density lipoprotein*.

**Figure 1:**
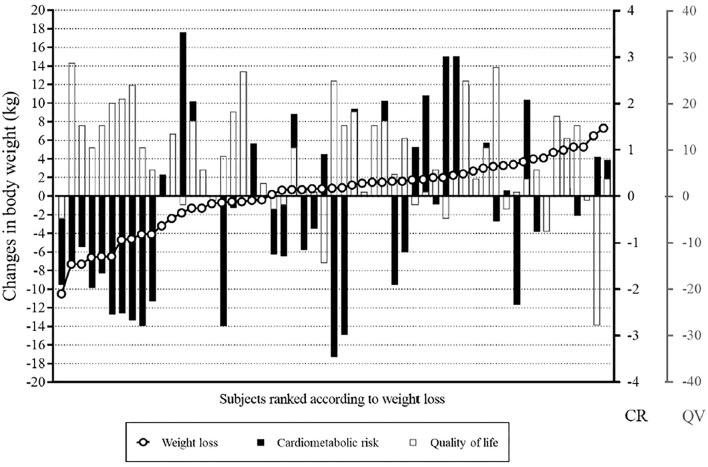
Individual data for changes in body weight, clustered cardiometabolic risk, and quality of life. Although the participants who lost more weight showed greater health improvements (those on the left side), benefits in cardiometabolic risk and quality of life can be seen all across the spectrum of changes in body weight. CR, cardiovascular risk: negative values mean improvement; QL, quality of life: positive values mean improvement.

**Figure 2:**
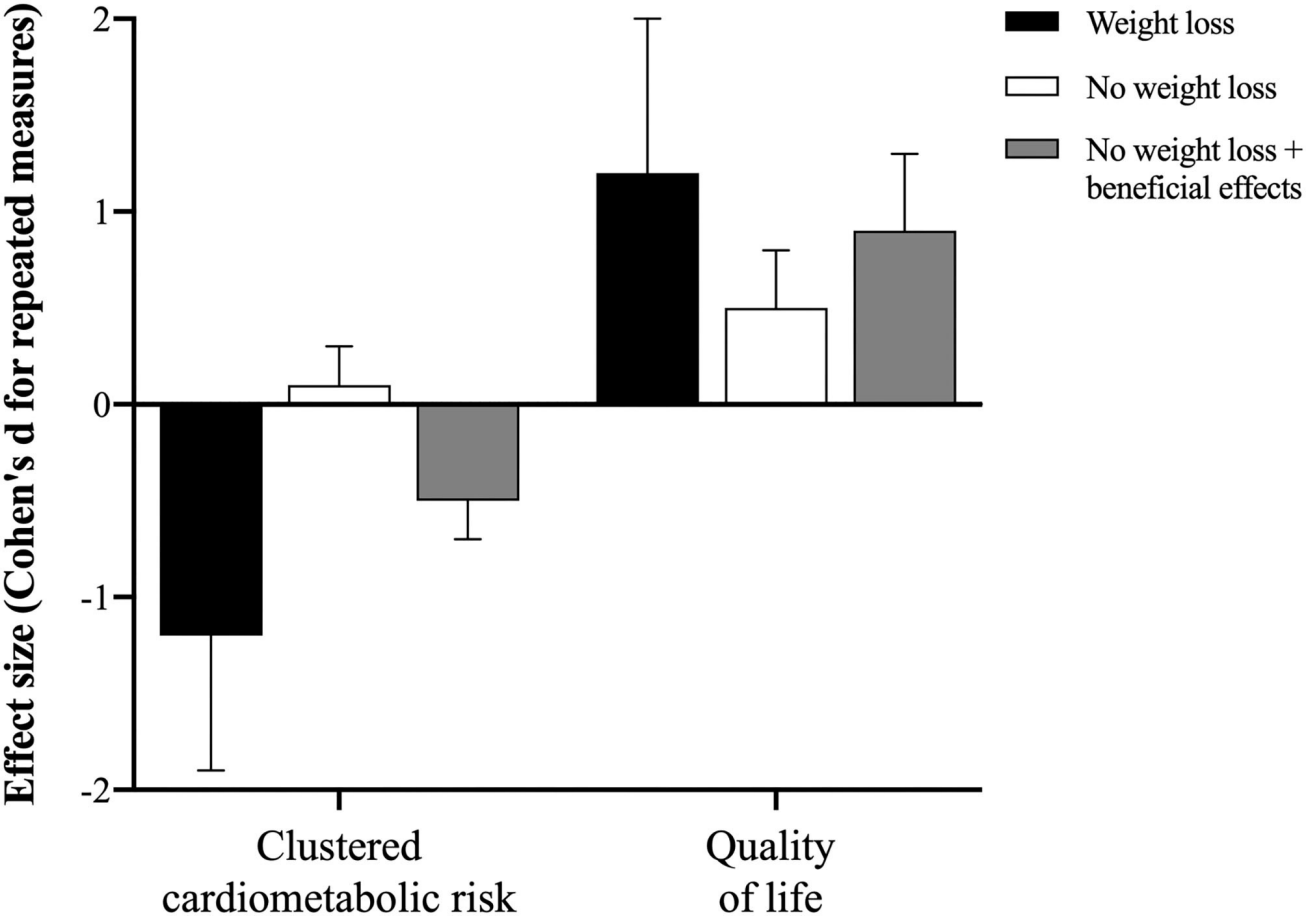
Effect size (Cohen's d for repeated measures) for clustered cardiometabolic risk, and quality of life in participants who lost weight (“weight loss”; *n* = 11), those who maintained or gained body weight (“no weight loss”, *n* = 44), and those who maintained or gained body weight and showed some improvement in cardiometabolic profile or quality of life (“no weight loss + beneficial effect”, *n* = 15 and 32, respectively). This overview picture supports the conclusion that intervention-induced weight loss induces greater improvements in cardiometabolic health; however, improvements in health and wellness may take place in the absence of weight loss or even weight gain, although the magnitude of the benefits is clearly lower, as compared to that of the first scenario.

Figure 1 illustrates individual data for changes in body weight, clustered cardiometabolic risk, and quality of life. All participants but one who reduced body weight (*n* = 11) consistently reduced clustered cardiometabolic risk (ES: −1.2, 95%CI: −1.9, −0.6) and improved quality of life (ES: 1.2, 95%CI: 0.5, 2.0). The magnitude of the changes in clustered cardiometabolic risk (ES: 0.1, 95%CI: 0.0, 0.3) and quality of life (ES: 0.5, 95%CI: 0.2, 0.8) was lower in participants who maintained or increased body weight. Interestingly, however, 34% and 73% of these participants who did not lose weight experienced improvements in clustered cardiometabolic risk (ES: −0.5, 95%CI: −0.7, −0.2) and quality of life (ES: 0.9, 95%CI: 0.6, 1.3), respectively (Figure 2).

## Discussion

To our knowledge, this was the first study to investigate whether weight loss following HAES®-based interventions is associated with changes in waist circumference, cardiovascular risk factors, clustered cardiometabolic risk, and quality of life in women with obesity. Our main finding was that weight loss was associated with improvements in selected cardiovascular risk factors, clustered cardiometabolic risk, and quality of life as expected; however, the majority of participants who maintained or even gained weight also benefited from the intervention to some extent.

Intentional weight loss is associated with reduction of all-cause mortality (16). In our study, weight loss associated with improvements in glucose, LDL, clustered cardiometabolic risk, and quality of life. Participants who lost weight were the ones who most benefited from the intervention, which is in line with the evidence that improvements in cardiovascular risk factors are proportional to the degree of weight loss (17). Indeed, weight loss is considered the most common target for success in obesity management. However, health-related benefits associated with weight loss may be better explained by concomitant reductions in total body and visceral fat, which are more strongly associated with cardiovascular risk than BMI itself (18).

The result showing that weight loss correlates with improvements in overall health following a lifestyle intervention is not novel. Nonetheless, our most striking finding was that some participants who maintained weight or even gained weight also improved waist circumference, clustered metabolic risk, and quality of life, with ES varying between moderate to high (although at values below those found for the participant who lost weight; see Figure 2 for an overview). These results corroborate the potential of eating and exercise interventions in improving health- and wellness-related markers to some level despite weight loss (4), and extend this notion to HAES®-based interventions, which refrain from targeting weight loss as a primary focus. In the majority of HAES®-based interventions (19, 20), physical activity is not an effective component of the intervention; despite participants are generally encouraged to practice physical activity, this is not formally included in the programs or even assessed as an outcome. Conversely, in our study, we developed a specific physical activity program based on HAES® approach, which is thoroughly described elsewhere (8). Indeed, the applicability of our program in different contexts (e.g., distinct sociocultural status, ages, body sizes, men groups, etc.) requires validation.

It has been argued that HAES® approach may lead to poor nutritional choices and to a state of passivity, resulting in weight gain (21). Our data challenge this notion by showing that 80% of our participants who underwent a weight-neutral intervention reduced or maintained weight. Notably, the participants also improved eating attitudes, body image, physical capacity, and quality of life (8, 9). The excessive focus on weight loss may deviate the focus on overall health gains potentially attained with lifestyle-modification programs characterized by an increase in physical activity and healthy eating (2, 4). Moreover, interventions highly centered in weight loss have been shown to lead to frustration due to weight loss failures (2), and reinforcement of fat stigma, according to which certain types of body are simply “inadequate”, potentially leading to body image and eating disorders (22). Our findings support the notion that interventions aimed at preventing obesity should be primarily focused on lifestyle-based behavior changes rather than weight loss, which should not be sole indicator of success in the management of obesity (4). Long-term studies should confirm the feasibility and efficacy of this sort of intervention, since obesity is a complex condition whose successful management relies in numerous biological, social and environmental factors.

In conclusion, improvements in cardiovascular risk factors and quality of life following an HAES®-based intervention were associated with weight loss. Indeed, beneficial effects were more pronounced in those who reduced body weight; however, participants who maintained or even gained weight also experienced benefits to some extent regarding cardiovascular health and quality of life. These findings suggest that weight loss enhances, but not determine, the beneficial effects of a weight-neutral, lifestyle-modification intervention, which can be an efficient strategy in the management of obesity.

## Data Availability Statement

The original contributions presented in the study are included in the article/supplementary material, further inquiries can be directed to the corresponding author/s.

## Ethics Statement

The studies involving human participants were reviewed and approved by Ethics Committee of the School of Public Health, University of São Paulo (protocol 1.738.855). The patients/participants provided their written informed consent to participate in this study.

## Author Contributions

MD, AP, FS, and BG conceived the presented idea. PM, FB, PL, DC, OR, FS, IP, LA, AV, and RF contributed to the design and implementation of the research. GS and MR contributed to the data analysis. All authors contributed to the analysis of the results and to the writing of the manuscript.

## Funding

This work was supported by the Research Support Foundation of the State of São Paulo (FAPESP), Grant Number 2015/03878-2. Finally, each author received a fellowship grant. FS was supported by CNPq (Grant Numbers 311357/2015-6 and 309514/2018-5) and FAPESP (Grant Number 2017/17424-9); AP, PM, and RF by FAPESP (Grant Numbers 2015/26937-4, 2017/05651-0, and 2015/12235-8, respectively); BG has a productivity grant by CNPq and is also supported by the Coordenação de Aperfeiçoamento de Pessoal de Nível Superior—Brasil (CAPES), and MD by CAPES—Finance code 001. The funding sources had no involvement in study design and in the collection, analysis and interpretation of data.

## Conflict of Interest

The authors declare that the research was conducted in the absence of any commercial or financial relationships that could be construed as a potential conflict of interest.

## Publisher's Note

All claims expressed in this article are solely those of the authors and do not necessarily represent those of their affiliated organizations, or those of the publisher, the editors and the reviewers. Any product that may be evaluated in this article, or claim that may be made by its manufacturer, is not guaranteed or endorsed by the publisher.
